# PathNER: a tool for systematic identification of biological pathway mentions in the literature

**DOI:** 10.1186/1752-0509-7-S3-S2

**Published:** 2013-10-16

**Authors:** Chengkun Wu, Jean-Marc Schwartz, Goran Nenadic

**Affiliations:** 1 Faculty of Life Sciences, University of Manchester, Manchester, M13 9PT, UK; 2 Doctoral Training Centre in Integrative Systems Biology, University of Manchester, 131 Princess Street, Manchester M1 7DN, UK; 4 School of Computer Science, University of Manchester, Manchester M13 9PL, UK; 3 Manchester Institute of Biotechnology, 131 Princess Street, Manchester M1 7DN, UK

**Keywords:** Biological pathway mentions, text mining, Alzheimer's pathways, systems biology

## Abstract

**Background:**

Biological pathways are central to many biomedical studies and are frequently discussed in the literature. Several curated databases have been established to collate the knowledge of molecular processes constituting pathways. Yet, there has been little focus on enabling systematic detection of pathway mentions in the literature.

**Results:**

We developed a tool, named PathNER (**Path**way **N**amed **E**ntity **R**ecognition), for the systematic identification of pathway mentions in the literature. PathNER is based on soft dictionary matching and rules, with the dictionary generated from public pathway databases. The rules utilise general pathway-specific keywords, syntactic information and gene/protein mentions. Detection results from both components are merged. On a gold-standard corpus, PathNER achieved an F1-score of 84%. To illustrate its potential, we applied PathNER on a collection of articles related to Alzheimer's disease to identify associated pathways, highlighting cases that can complement an existing manually curated knowledgebase.

**Conclusions:**

In contrast to existing text-mining efforts that target the automatic reconstruction of pathway details from molecular interactions mentioned in the literature, PathNER focuses on identifying specific named pathway mentions. These mentions can be used to support large-scale curation and pathway-related systems biology applications, as demonstrated in the example of Alzheimer's disease. PathNER is implemented in Java and made freely available online at http://sourceforge.net/projects/pathner/.

## Background

Systems biology places special emphasis on the large-scale integration of various data and "scattered pieces of knowledge" [1]. This is particularly challenging for the knowledge coming from the literature, which is one of the most important sources of information for biological studies. Over 20 million citations have been included in PubMed [2] and this number is growing. Various text mining techniques have been explored, developed and used to provide access to biological knowledge from that massive amount of publications in a systematic and automated way to support knowledge discovery and hypothesis generation in particular in the context of systems biology [3].

Biological Named Entity Recognition (NER), specifically, is a key part of biomedical text mining as biological entities are basic actors in biological systems and processes. Promising and useful results have been achieved in recognising genes [4], chemicals [5], anatomy parts [6], species [7], etc. Alongside with those entities, biological pathways play an important role in systems biology studies. Each pathway is formed by a collection of entities (e.g. proteins) and interactions, and carries some biological function. For many pathways, specific names are gradually established and used within the community in order to avoid repeated descriptions of molecular details for the convenience of communication. Yet, there has been little focus on enabling systematic detection of pathway mentions in the literature. On the other hand, similarly to entities and molecular interactions, biological pathways are extensively curated. A number of databases have been built to store the details of curated pathways, including KEGG [8], Pathway Interaction Database (PID) [9], WikiPathways [10], MetaCyc [11], Reactome [12] and integrative databases like Pathway Commons [13] and ConsensusPathDB [14]. ConsensusPathDB in particular is one of the most comprehensive collections of pathways as it integrates more than 4,000 pathways from twelve data sources (see Table 1). Furthermore, the Pathway Ontology (PO) [15] is built to organise various types of biological pathways including classic metabolic, regulatory, signalling, drug and disease pathways. It also offers altered pathways and lists of pathway name synonyms.

**Table 1:** Number of pathway entries in different data sources

Data source	Number of pathways*
PID	1478
Reactome	1326
WikiPathways	423
SMPDB	411
HumanCyc	305
KEGG	257
Biocarta	254
Pharmgkb	97
INOH	93
EHMN	69
NetPath	26
Signalink	15

*Numbers are based on ConsensusPathDB, accessed on 30/04/2013

Although the so-called "deep approach" to pathway curation is likely to remain dominant in the near future as automated methods for pathways reconstruction are yet to match human curators [1], several text-mining applications have been used to support automatically (re)constructing pathways from molecular interactions and metabolic reactions described in publications [16-18] (see [19] for an overview). For example, PathText [18] integrates pathway visualisation, text mining and annotation tools, and supports navigation through pathways where nodes and links are enriched by text mining results. The objectives of those pathway reconstruction systems are different from the focus of this paper: while they are aiming for automated extraction of molecular details to build pathways, we focus on the recognition of mentions of known pathways in the literature. To achieve that, we developed PathNER, a NER tool dedicated to the systematic identification of named pathway mentions in the literature using text-mining techniques. It aims to support both systems biology studies and pathway curation efforts. For example, the identification of pathway mentions in the literature makes it possible to answer questions of the type "what pathways have been linked to a given disease?". Furthermore, the analysis of pathway mentions could provide valuable hints on pathways that should be prioritised for curation. In addition, pathways that are frequently reported as associated to the disease of interest could be the focus of community efforts towards consensus descriptions (see [20] for an example).

There are several systems that are closely related but not dedicated to the task presented here. For example, LitInspector [21] labels genes and general pathway keywords in PubMed abstracts to improve their readability and accelerate literature search. Its support for mining signal transduction pathways is however limited to exact matching of a proprietary list of names and keywords. CoPub [22] is a general term search system that also supports identification of pathway terms defined by a thesaurus compiled from KEGG, HumanCyc and Reactome. The search in CoPub is empowered by regular expression matching with some limited expansion of exact dictionary matching of its pathway thesaurus. PathNER, on the other hand, uses soft dictionary matching and flexible rules to identify potential pathway mentions.

In the remainder of this paper, we first introduce PathNER and evaluate its performance on a gold-standard corpus we developed. We then demonstrate the potential of PathNER in a case study focused on pathways related to Alzheimer's disease.

## Methods

We focus on named pathway mentions that are referred in text by using a specific name (e.g. "MAPK signalling pathway", "Calcium signalling"). Such names often contain general pathway-specific keywords ("pathway", "signalling pathway", "cascade", etc.) and mentions of associated genes. However, many occurrences of general pathway keywords are ambiguous (e.g., "the diagnostic pathway" is not a biological pathway). Inversely, many pathway names do not contain obvious pathway-related keywords (e.g. "RNA degradation" [KEGG PATHWAY: hsa03018]).

### Dictionary of pathway names

A dictionary of pathway names was compiled from the Pathway Ontology [15] and ConsensusPathDB [14], focusing on human biological pathway mentions only. We generated additional variants from entries that contained acronyms or synonyms. The final dictionary contains 6,343 entries (see Additional file 1). Each entry is composed of a pathway identifier (ID) and a pathway name. Pathway IDs contains information about the source database.

We note that there are some general names in the dictionary, such as "Disease", "Metabolism" and "Gene Expression". Those mentions mostly come from Reactome and KEGG. We decided to keep all entries for completeness and automated updates, although we note that they are ambiguous and can be used to describe generic biological concepts.

To better understand the composition of pathway names, we have conducted a lexical profiling of six representative databases (BioCarta, KEGG, PID, Reactome, WikiPathways, Pathway Ontology). We analysed frequent tokens and keywords and mentions of genes/proteins. The analysis revealed some interesting (but expected) differences between different pathway database sources (see the Results section).

### PathNER workflow

**Figure 1:**
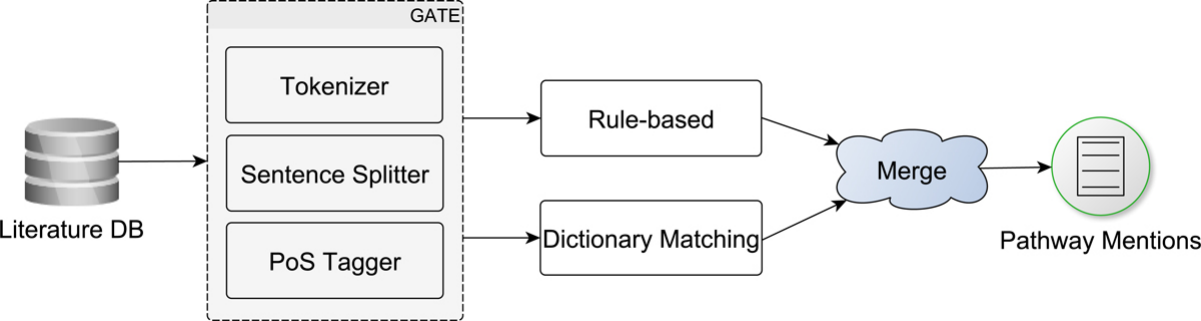
**Workflow of PathNER**. PathNER is built upon GATE framework and combines soft dictionary matching and rule-based detection.

The workflow implementing PathNER is shown in Figure 1. PathNER applies soft dictionary matching and rules. Pathway mentions identified by the two components are consequently merged. PathNER is built upon the open-source text engineering framework GATE Embedded [23]. Documents are pre-processed (tokenisation, sentence splitting, Part-of-Speech (POS) tagging) using the OpenNLP plugin.

### Soft dictionary matching

To match pathway mentions in text, we employed soft dictionary matching as term variations often cause exact string matching to fail [24]. Typical term variations include insertion/deletion of stop-words and word permutations (often due to the use of prepositions, e.g. "p53 signalling pathway" and "signalling pathway of p53"), morphological variations (e.g. "transcriptional factor" and "transcription factor") and using alternative keywords (e.g. "MAPK pathway" and "MAPK signalling pathway").

The core of the soft dictionary matching method is calculating the similarity between two given strings (one as query, the other as pattern) using appropriate distance metrics. PathNER utilises the SoftTFIDF method implemented in the open-source Java toolkit SecondString [25]. SoftTFIDF is a combination of the TFIDF weighting scheme and the Jaro-Winkler string distance scheme, and has been reported as one of the best performing among multiple commonly seen string distance metrics [25]. The SoftTFIDF dictionary lookup takes a dictionary and a query string as input, and outputs a list of possible matches ranked by their SoftTFIDF similarity score (a float number between 0 and 1) against the query string. A configurable threshold is used to filter low-scoring matches.

To match against a whole document, a set of candidate query strings needs to be generated. We have developed a tailored method where the first step for candidate selection is to find possible starting points for potential candidates. To that end, each token is first sent to SoftTFIDF lookup configured with a relatively low threshold (any match scores less than this threshold will be discarded at this stage; the value of this threshold (referred to as "the lower threshold") is empirically set as 0.40). If the result set is not empty, then the token's position is recorded as a potential starting point for a possible match candidate. This prevents unnecessary matching attempts starting from tokens that do not appear in the dictionary. In the second step, we check each recorded starting point with different lengths of the window: each check starts with a window length of one and the window length is increased until it reaches the maximum (the longest entry in the dictionary has 20 tokens, but the maximum was set to be 25 to allow potential insertion of stop-words). Tokens within the window form a query string for SoftTFIDF that is also configured with the lower threshold. If the result set is not null, the check continues; if more than four (an empirically determined value) consecutive window lengths find no matches by SoftTFIDF lookup, the check stops and a new check starts from the next possible position. During each check, the match results for different window lengths are stored. The longest possible match which scores over a high threshold (empirically set as 0.90) are returned. Generally speaking, a smaller value for the lower threshold slows down the processing speed, whereas a larger value can decrease recall; for the higher threshold, a greater value potentially increases precision but reduces recall and a smaller value increases recall but potentially hinder precision. We note that these thresholds are configurable in PathNER.

### Rule-based recognition

The rule-based component aims to complement the soft dictionary lookup method in particular to address name variation and missing names. It works by integrating three types of information: general pathway-specific keywords, POS tags and gene/protein recognition results. The component is implemented using the Java Annotation Patterns Engine (JAPE) within the GATE framework.

The pathway-specific keywords we used include "pathway", "signalling" (and "signalling"), "transduction", "cascade" and "network". Specific combinations of those keywords are also considered as keywords (e.g., "signalling pathway", "signalling cascade", "signalling network", etc.). Gene/protein mentions are recognised by BANNER [4].

We developed two types of rules: backward and forward. Backward rules start from a keyword found in the text. PathNER then scans backwards for one of the following five types of tokens: determiners (e.g. "the", "all", "such", etc.), separators (e.g. comma, period, semicolon, etc.), prepositions or subordinating conjunctions (e.g. "about", "in", "because", "after", etc.), wh-words (e.g. "when", "whatever", etc.) and verbs. These tokens indicate potential starting points of a pathway mention. Forward rules scan forward from the pathway keyword position for pathway-specific verb phrases (e.g. "induced by", "regulated by", "mediated through", etc.) until any punctuation character is met.

Segments anchored by these rules are then integrated with the results generated by BANNER. If BANNER reports any mention of gene/protein-like names that are contained within a rule-detected segment, then the segment is tagged as a named pathway mention.

### Results merging

We generate the final annotation set by merging the results from the above two components. Mentions detected by only one component are added directly to the final set. For overlapped annotations, a new annotation covering both is generated and added to the final set.

### Evaluation Methodology

There is no gold-standard corpus that annotates pathways at the mention level. We used the GENIA corpus [26] to generate a subset of abstracts with manually annotated pathway mentions. The original GENIA corpus contains 1,999 Medline abstracts, which were retrieved from PubMed using three MeSH terms "human", "blood cells" and "transcription factors". The terms in the corpus have been manually annotated for over 30 biological entity types including proteins, genes and cells. Although pathways are not specifically included in original GENIA annotations, we have noted that many of pathway mentions have been annotated with a generic label (G#other_name).

Our gold-standard corpus was derived from GENIA in two parts, which are both manually annotated for mentions of named pathways. The first part is a collection of 50 randomly sampled abstracts from the GENIA corpus. As randomly selected articles might contain too few pathway mentions for meaningful evaluation, we engineered the second part to ensure that there are adequate pathway mentions for evaluation. This part is derived from the GENIA sentences that contain both a G#other_name label and pathway-specific keywords. These sentences were manually checked and only named pathway mentions were labeled. As a result, the annotated gold-standard corpus (available in Additional file 2) contains 726 annotations of pathway mentions, of which 448 are lexicographically unique. We note that 379 pathways appeared only once in the corpus.

To measure the performance of PathNER against the gold-standard corpus, we adopted standard metrics: Precision (P), Recall (R) and F1-score (F1) defined by the following equations:

$$P=\frac{TP}{TP+FP},\;R=\frac{TP}{TP+FN},\;F1=2\frac{P\cdot R}{P+R}$$

where *TP *is the number of *true positives*, *FP *is the number of *false positives *and *FN *is the number of *false negatives*.

### Application of PathNER on Alzheimer's disease corpus

To illustrate the potential of PathNER for curators and systems biologists who are interested in relationships between pathways and diseases, we applied PathNER to Alzheimer's disease (AD). AD-related pathways have been already manually curated within the AlzPathway database [27]. The database contains 119 intra, inter and extra cellular AD signalling pathways curated from over 100 review articles. The curation process followed a two-step procedure: the first step determined what pathways are involved in AD, whereas the second step retrieved molecular details for those selected pathways. We aim to illustrate here how PathNER can be used to support the first step.

To support the task, we constructed two corpora of AD-related articles: Alz_ARF_PubMed and Alz_ARF_PMC (see Table 2 and Additional file 3). Alz_ARF_PubMed contains abstracts and comes from the recommendations of the Alzheimer Research Forum (ARF) [28]. Alz_ARF_PMC contains available full-text articles of abstracts from Alz_ARF_PubMed; only open-access articles were retained for text mining. Documents have been downloaded using *gnEutils4j *[29]. After applying PathNER on these corpora, we manually normalised pathway mentions to get unique mentions (for instance, "Wnt signalling cascades", "Wnt-signalling", "wnt signal transduction pathway", "wingless-type signalling" etc. were all normalised to "'Wnt signalling pathway").

**Table 2:** Statistics of the AD corpora

Corpus	Type	# of articles	# of open-access
Alz_ARF_PubMed	Abstracts	1,983	ALL
Alz_ARF_PMC	Full-texts	732	87

## Results

### Dictionary profiling

Table 3 shows lexical profiles (top 10 tokens) of six representative databases (BioCarta, KEGG, PID, Reactome, Wikipathways, Pathway Ontology). It is interesting that the table highlights the different foci of the databases. For instance, "metabolism" comes on top for KEGG, whereas others databases contain more occurrences of "pathway" and "signalling" keywords. The differences in lexical constituents highlight the importance of compiling different entries into the dictionary.

**Table 3:** Top 10 tokens from pathway names in representative databases

	BioCarta		KEGG		PID		Reactome		WikiPathways		PO	
**Rank**	**Token**	**Freq**	**Token**	**Freq**	**Token**	**Freq**	**Token**	**Freq**	**Token**	**Freq**	**Token**	**Freq**

#1	pathway	6.09%	metabolism	6.01%	signalling	3.66%	activation	1.68%	signalling	5.38%	pathway	23.34%
#2	signalling	4.63%	pathway	3.63%	pathway	2.56%	signalling	1.63%	pathway	4.55%	signalling	9.52%
#3	regulation	1.79%	signalling	3.50%	activation	1.27%	metabolism	1.18%	metabolism	3.11%	altered	3.02%
#4	cell	1.65%	biosynthesis	3.25%	events	1.23%	synthesis	1.06%	regulation	1.38%	metabolic	2.88%
#5	role	1.06%	cell	1.50%	regulation	1.17%	regulation	0.95%	cell	1.31%	mediated	1.69%
#6	receptor	1.06%	acid	1.38%	mediated	1.03%	mediated	0.90%	receptor	1.04%	biosynthetic	1.39%
#7	activation	0.99%	cancer	1.25%	receptor	1.02%	transport	0.86%	activity	0.83%	degradation	0.79%
#8	kinase	0.86%	infection	1.00%	cell	0.73%	receptor	0.80%	synthesis	0.83%	drug	0.78%
#9	gene	0.73%	disease	0.88%	metabolism	0.64%	complex	0.69%	cycle	0.83%	factor	0.78%
#10	cycle	0.66%	degradation	0.75%	synthesis	0.63%	receptors	0.69%	proteins	0.83%	acid	0.72%

Table 4 shows the number of entries that contain general pathway-specific keywords and gene/protein names (as detected by BANNER). Seven out of twelve databases analysed have more than half of their entries contained mentions of genes/proteins and keywords. We note that this percentage could be larger as BANNER failed to recognise gene/protein names in several entries, as it requires a wider context for processing than just a pathway name string. It is therefore important that our rule-based component takes gene/protein names and keywords into account.

**Table 4:** Numbers of dictionary entries with pathway keywords and gene/protein names

Database	#Total entries	#Entries with keywords	#Entries with keywords and gene/protein	% with gene/protein in entries with keyword
INOH	90	55	53	96.36%
NetPath	48	48	45	93.75%
PID	1329	433	280	64.67%
KEGG	273	39	22	56.41%
WikiPathways	376	129	72	55.81%
Reactome	1411	243	134	55.14%
BioCarta	260	117	64	54.70%
PO	1609	1556	512	32.90%
PharmGKB	96	92	17	18.48%
SMPDB	467	215	33	15.35%
HumanCyc	308	29	3	10.34%
EHMN	78	1	0	0.00%

### PathNER performance on gold corpus

We separately evaluated the performance of PathNER components and the integrated system. The results for both strict (requires exact matching between annotations by the gold standard and annotations by PathNER) and lenient matching (annotations by PathNER that overlap with the gold standard are accepted) are presented in Table 5. PathNER achieves a lenient F1-score of 84%. The combination of soft dictionary matching and rules considerably improved the performance. For lenient evaluation, recall improved from 58% (rule-based) and 63% (soft dictionary matching) to 88% (hybrid), while keeping precision at an acceptable level (81%). This is similar in strict evaluation: recall improved from 51% (rule-based) and 44% (dictionary matching) to 80% (hybrid), with precision at 74%. For comparison, a baseline method that used our dictionary and dictionary matching typically used for NER (we used LINNAEUS [7]) achieved notably lower values (F1-score of 38% and 54% for strict and lenient matching respectively).

**Table 5:** Performance evaluation of PathNER

Method	Strict			Lenient		
	Recall	Precision	F1-score	Recall	Precision	F1-score
Baseline	0.32	0.49	0.38	0.43	0.66	0.54
Soft dictionary	0.44	0.51	0.47	0.63	0.72	0.67
Rules	0.51	**0.86**	0.64	0.58	**0.97**	0.72
PathNER	**0.80**	0.74	**0.77**	**0.88**	0.81	**0.84**

### PathNER results on the AD corpora

Table 6 lists the number of detected mentions in the AD corpora. Each full-text article contains around ten pathway mentions on average, among which around two are unique. This is much higher than what can be found in abstracts (less than 0.2 mentions per abstract), so the availability to process full-text articles is extremely important.

**Table 6:** Numbers of pathway mentions in the AD corpora

Corpus	Processed articles	Total mentions	Unique mentions	Mention per article	Unique mention per article
Alz_ARF_PubMed	1,983	1,961	363	0.99	0.18
Alz_ARF_PMC	85	883	203	10.39	2.39

Table 7 and Table 8 further show the top 25 most frequent pathway mentions detected in the AD corpora. The different corpora exhibit quite good consistency (19 shared mentions in the top 25) but the differences again highlight the need to process full texts. For instance, the "Insulin/IGF-1 Signalling" detected from Alz_ARF_PMC is not in the top 25 of Alz_ARF_PubMed but it is indeed closely related to AD [30].

**Table 7:** Top 25 detected mentions in the ALZ_ARF_PUBMED corpus

Detected Mention	Freq	In AlzPathway?	Evidence
Alzheimer's disease	1869	N/A	N/A
Disease	1121	N/A	N/A
Parkinson's disease	201	NO	PMID: 12672864
Amyotrophic lateral sclerosis	143	NO	PMID: 1571856
Metabolism	123	N/A	N/A
Apoptosis	120	YES	PMID: 11227497
Oxidative stress	99	YES	PMID: 10681270
Transcription	99	N/A	N/A
Long-term potentiation	98	YES	PMID: 12399581
Gene expression	94	YES	N/A
Proteasome	67	NO	PMID: 10854289
Huntington's disease	59	NO	PMID: 15686606
Cell cycle	56	YES	PMID: 15936057
Methylation	35	NO	PMID: 19606065
Translation	35	N/A	N/A
Acetylation	33	YES	PMID: 19625751
Endocytosis	27	NO	PMID: 16442855
Notch signalling	21	NO	PMID: 19853579
Glucose metabolism	18	NO	PMID: 21971455
Obesity	17	NO	PMID: 19801534
Long-term depression	16	NO	PMID: 21854392
Signal transduction	15	N/A	N/A
Glycolysis	14	NO	PMID: 14718371
Prion diseases	14	NO	PMID: 15190676
Creutzfeldt-Jakob disease	13	NO	PMID: 7904883

*N/A: Not applicable; the last column shows a PMID that provides evidence that a given mention is linked to AZ.

**Table 8:** Top 25 detected mentions in the ALZ_ARF_PMC corpus

Detected Mention	Freq	In AlzPathway?*	Evidence
Disease	635	N/A	N/A
Alzheimer's disease	174	N/A	N/A
Amyotrophic lateral sclerosis	130	NO	PMID: 1571856
Methylation	95	NO	PMID: 19606065
Long-Term Potentiation	78	YES	PMID: 12399581
Oxidative Stress	69	YES	PMID: 10681270
Transcription	65	N/A	N/A
Parkinson's Disease	48	NO	PMID: 12672864
Cell cycle	46	YES	PMID: 15936057
Metabolism	44	N/A	N/A
Axon guidance	32	YES	PMID: 17571925
Gene expression	31	YES	N/A
Glucose metabolism	23	NO	PMID: 21971455
Calcium signalling	20	YES	PMID: 21184278
Acetylation	18	YES	YES
Apoptosis	16	YES	PMID: 11227497
Activation of the Rac signalling pathway	15	NO	PMID: 10817927
Notch signalling	14	NO	PMID: 19853579
Prion diseases	12	NO	PMID: 15190676
Proteasome	12	NO	PMID: 10854289
S phase	12	YES	PMID: 19946466
Translation	12	N/A	N/A
Endocytosis	10	NO	PMID: 16442855
Insulin/IGF-1 Signalling	10	NO	PMID: 22817723
Post-translational modifications	9	YES	PMID: 21215781

*N/A: Not applicable; the last column shows a PMID that provides evidence that a given mention is linked to AZ.

It is interesting that only six and ten out of 25 top mentioned pathways in the respective abstract and full-text corpora already appeared in the AlzPathway database. We however note that AlzPathway focuses on signalling pathways, whereas PathNER also extracted a number of metabolic pathways. Still, the Notch signalling, which was identified by PathNER, does not appear in AlzPathway, although it has been reported as important for understanding the pathogenesis and treating of AD [31]. To further validate the results, we searched for literature evidence for each non-curated pathway in the top 25 - none of those was irrelevant to AD (see tables 7 and 8 for relevant evidence publication).

There are, on the other hand, pathways present in AlzPathway that were not detected by PathNER. One of the likely reasons is that PathNER was applied on a limited set of articles (less than 2,000; PubMed returns more than 5,400 abstracts related to AD that mention any of the pathway-specific keywords).

## Discussion

PathNER is based on soft dictionary matching and additional rules, which enables it to address term variations. The performance improvement shows the complementarity of the two approaches. The rules, in particular, proved to be useful for spotting complex pathway mentions. For example, "*...activation of the ras/raf/MAPK kinase (MEK)/ERK and phosphatidylinositol 3-kinase (PI-3K)/AKT/mammalian target of rapamycin (mTOR) signalling pathways*" [PMID: 19723757] can be captured by PathNER but it can be very hard for any dictionary-based method. While the rules were often successful, their coverage can still be improved. For instance, PathNER was not able to recognise a much simpler "microglial activation" (which is a pathway listed in AlzPathway) as it did not contain any of our pathway-specific keywords or gene/protein name. PathNER would therefore need to be extended with other potential clues. For example, Table 4 shows that the percentage of gene/protein names is low in names of pathways appearing in PharmGKB (mainly about drug pathways), SMPDB (small molecule pathways), HumanCyc and EHMN (mostly about metabolic pathways), and therefore other specific keywords need to be identified. In addition, some false positives were due to missed gene/protein names, so integration with other gene name recognition tools might be useful (e.g. [32], [33]). Similarly, context-dependent identification of acronyms and their expended forms would help in detecting mentions such as "T cell receptor (TCR) signalling".

If recognised pathway mentions are to be linked to a database entry, then rules will need to provide some additional matching. For instance, although 'the CD2 pathway' has been mentioned in many articles related to T lymphocytes, it was not an entry in our dictionary. CD2 is the acronym for "Cluster of Differentiation 2" and it represents a cell adhesion molecule. The closest relevant entry we found was "Cell adhesion molecules (CAMs)" [KEGG:hsa04514], but linking "the CD2 pathway" to "Cell adhesion molecules (CAMs)" would require significant background knowledge.

The AlzPathway case study was used to illustrate the potential of PathNER in identifying relationships between pathways and disease, using a relatively small set of documents. Even within this restricted scenario, we have shown how PathNER can help identity relevant disease pathways, and thus facilitate the curation process. It generated some new suggestions that were not included in the human-curated AlzPathway database. PathNER could provide a curation support by enabling fast detection of pathway mentions based on a much more comprehensive collection of articles than used in AlzPathway (only 100 articles) or indeed in our case study (around 2000 articles). The results are interesting in two aspects: on one hand, PathNER could contribute to prioritising pathways for curation (e.g. most frequent ones; most recent or most cited) and thus help address the limitations on time and resources available to carry out the curation. On the other hand, the successful application of PathNER on the AD corpora suggests that PathNER can be used to guide the curation of pathway maps related to other human diseases in a systematic way (e.g. the diseases where there are no organised community curation efforts).

PathNER integrates dictionaries and rules, instead of relying on machine learning which is another popular NER approach. There are several reasons for that. Machine-learning approaches require a fairly large amount of annotated samples for training, which were not available in this case. On the other hand, public pathway databases with names are readily available and patterns commonly used for mentioning pathways are well understood. Therefore, an approach based on soft dictionary matching and rules becomes a natural choice.

As a NER tool, PathNER aims to identify pathway name mentions. On the other hand, text-mining systems that aim to reconstruct pathways from literature aim to identify constituent entities in the text and then perform information extraction to identify the relationship between those entities. Consequently, we cannot compare PathNER with those systems as they are addressing different problems. However, PathNER can serve as a complementary/component tool in such systems as different entities (genes, proteins, small molecules, etc.) can interact with pathways to build new pathways. By tagging pathway mentions as a type of entity with PathNER, it would be possible to utilise existing interaction extraction systems (e.g. [34]) to retrieve interactions that involve named pathways that contribute to the pathogenesis of certain diseases.

## Conclusions

In this paper we presented PathNER, a tool that can systematically detect biological pathway mentions in literature with a lenient F1-score of 84%. In contrast to existing text-mining efforts that target the automatic reconstruction of pathway details from molecular interactions mentioned in the literature, PathNER focuses on identifying specific named pathway mentions. We also demonstrated that PathNER could provide a curation support by providing reliable curation suggestions from most frequent pathway mentions in the primary literature associated to a particular disease. Another potential audience of PathNER are systems biologists who want to investigate the relationship between pathways and disease or other biological entities. The future work will include extending the rule component, in particular through mapping of recognised mentions to most similar entries in pathway databases. In addition, we aim to develop a "pathway names crawler" that will crawl the literature for new pathway names to update our dictionary.

## Availability

PathNER together with the Alzheimer's corpora as well as the extracted results are available at http://sourceforge.net/projects/pathner/ under the Common Public License 1.0.

## List of abbreviations used

POS: Part-Of-Speech; NER: Named Entity Recognition; AD: Alzheimer's disease; KEGG: Kyoto Encyclopaedia of Genes and Genomes; PID: Pathway Interaction Database; PO: Pathway Ontology; JAPE: Java Annotation Pattern Engine; PMID: PubMed ID.

## Competing interests

The authors declare that they have no competing interests.

## Authors' contributions

CW developed PathNER and deployed the detection on the Alzheimer's corpora, and drafted the manuscript. GN provided support and guidance from the text mining perspective and JMS from the systems biology perspective. GN and JMS conceived and supervised the project. CW, JMS and GN wrote the manuscript. All authors read and approved the final manuscript.

## Supplementary Material

Additional file 1 **Dictionary of pathway names**. The dictionary is a .txt file, with each line represents one dictionary entry. The data comes from ConsensusPathDB and Pathway Ontology. (human data only). Click here for file

Additional file 2 **Gold corpus**. The gold corpus is an .xml file. To open, please download and install the freely available GATE developer program [http://gate.ac.uk/]. After installation, open GATE, and create a new 'Language Resources' with the 'GATE Document' type, select the path of this .xml file. Click here for file

Additional file 3 **AD corpora**. The corpora are compressed in the .tar.gz file. Once decompressed, you will get four separate .txt files. Each txt file contains multiples lines of PubMed IDs or PubMed Central IDs, one ID per line. Click here for file

## Declarations

Publication of this article was funded by the UK Biotechnology and Biological Sciences Research Council (BBSRC) through the Doctoral Training Centre for Integrative Systems Biology at the University of Manchester.

This article has been published as part of *BMC Systems Biology *Volume 7 Supplement 3, 2013: Twelfth International Conference on Bioinformatics (InCoB2013): Systems Biology. The full contents of the supplement are available online at http://www.biomedcentral.com/bmcsystbiol/supplements/7/S3.
